# Biomarker analysis of American toad (*Anaxyrus americanus*) and grey tree frog (*Hyla versicolor*) tadpoles following exposure to atrazine

**DOI:** 10.1016/j.aquatox.2016.11.018

**Published:** 2016-11-21

**Authors:** Marcía N. Snyder, W. Matthew Henderson, Donna A. Glinski, S. Thomas Purucker

**Affiliations:** a Grantee to U.S. Environmental Protection Agency via Oak Ridge Institute of Science and Education, Athens, GA, 30605, United States; b U.S. Environmental Protection Agency, Office of Research and Development, National Health and Ecological Effects Laboratory, 200 SW 35th St., Corvallis, OR, 97333, United States; c U.S. Environmental Protection Agency, Office of Research and Development, National Exposure Research Laboratory, 960 College Station Road, Athens, GA, 30605, United States

**Keywords:** Biomarkers, Amphibians, Pesticides, Support vector machine, Metabolites

## Abstract

The objective of the current study was to use a biomarker-based approach to investigate the influence of atrazine exposure on American toad (*Anaxyrus americanus*) and grey tree frog (*Hyla versicolor*) tadpoles. Atrazine is one of the most frequently detected herbicides in environmental matrices throughout the United States. In surface waters, it has been found at concentrations from 0.04–2859 μg/L and thus presents a likely exposure scenario for non-target species such as amphibians. Studies have examined the effect of atrazine on the metamorphic parameters of amphibians, however, the data are often contradictory. Gosner stage 22–24 tadpoles were exposed to 0 (control), 10, 50, 250 or 1250 μg/L of atrazine for 48 h. Endogenous polar metabolites were extracted and analyzed using gas chromatography coupled with mass spectrometry. Statistical analyses of the acquired spectra with machine learning classification models demonstrated identifiable changes in the metabolomic profiles between exposed and control tadpoles. Support vector machine models with recursive feature elimination created a more efficient, non-parametric data analysis and increased interpretability of metabolomic profiles. Biochemical fluxes observed in the exposed groups of both *A. americanus* and *H. versicolor* displayed perturbations in a number of classes of biological macromolecules including fatty acids, amino acids, purine nucleosides, pyrimidines, and mono- and di-saccharides. Metabolomic pathway analyses are consistent with findings of other studies demonstrating disruption of amino acid and energy metabolism from atrazine exposure to non-target species.

## 1. Introduction

Multiple stressors are driving the global decline of amphibians including habitat loss, disease, climate change, and exposure to pesticides (Cushman, 2006; Wake, 2012). The impact of pesticides on amphibian health is among the least understood drivers of amphibian decline (Orton and Tyler, 2015). Predictions of adverse effects from pesticide exposure on amphibians are difficult to make with high certainty because results of laboratory toxicity studies are often inconsistent and even contradictory (Rohr and McCoy, 2010; Orton and Tyler, 2015; Solomon et al., 2008; Van Der Kraak et al., 2014). Often times a lack of understanding of the mechanism of action between a xenobiotic stressor and traditional toxicological effects endpoints (e.g. mortality, growth rate, population decline, etc.) exists; understanding early perturbations in biochemical pathways using ‘omics technologies will help inform these adverse outcome pathways.

Metabolomics is an analytical tool that can be used to capture changes in endogenous metabolites and an interpretation of these changes can be used as early predictions of the consequence of exposure to contaminants in non-target organisms such as amphibians. Concentrations of endogenous metabolites directly link the phenotype of the organism to its biochemical pathways and therefore may be measured before changes occur in other frequently assessed effects endpoints. Metabolomics can be used to determine biomarkers of exposure by examining an entire suite of potential pathways influenced versus measuring just one or two pre-determined markers such as glycogen or ATP (Dornelles and Oliveira, 2014; Zaya et al., 2011a; Patti et al., 2012). Metabolomics alone or coupled with genomics or proteomics have been successfully used to develop biomarkers of many environmental stressors as well as xenobiotics (Lin et al., 2014; Viant et al., 2006). Ultimately, an understanding of these exposure induced modifications in metabolomic pathways will facilitate identification of the toxic mode of action of pesticides which can be utilized to decrease the uncertainty of xenobiotic stress on amphibians.

Combining metabolomics with machine learning techniques allows for pattern classification that elucidates the link between exposure and toxic response by connecting the phenotypic state of the organism exposed with the underlying biochemical responses. Machine learning analysis is a powerful tool that is often used with multivariate data with large numbers of predictor variables. Metabolomics can create tens of thousands of data points for a single sample. One of the rate limiting steps in a metabolomics study is distinguishing the metabolomic stress response from un-induced biological variability in metabolites within the metabolomic profile. Support Vector Machine (SVM) analysis can classify non-linear high-dimensional data as is typically generated by metabolomic studies (Luts et al., 2010). SVM is designed to avoid over-fitting while classifying data with a large number of response variables. SVM combined with recursive feature elimination (RFE) can be used to ranks bins in the metabolomic profiles in order of importance, creating more efficient data analysis while increasing accuracy and ease of interpretability of the results (Heinemann et al., 2014).

We use atrazine as a case study to understand how metabolomics coupled with machine learning statistical tools can increase knowledge of how pesticides impact amphibian larvae. Non-target aquatic organisms have the potential to be exposed to atrazine because of its ubiquitous occurrence in surface waters. Atrazine is one of the most commonly detected pesticides in ground and surface waters throughout the United States. In a national-level survey of flowing waters draining agricultural, urban, or mixed land use areas, atrazine was detected more than 75% of the time (Stone et al., 2014). Atrazine is an herbicide that affects plants by inhibiting photosynthesis. The predominant use is on corn, sorghum, and sugarcane crops to control broadleaf plants, although it is also used with various other crops (Fernandez-Cornejo et al., 2014). It has been found at concentrations from 0.04–2850 μg/L in aquatic ecosystems, presenting a likely sub-lethal chronic exposure scenario for non-target species such as amphibians (U.S. EPA, 2016). Maximum 21-day average atrazine concentrations found in surface waters range from 0.01–233.57 μg/L have been measured in streams, lakes, and reservoirs as part of U.S. Geological Survey ambient water quality monitoring (Murphy et al., 2006; Thurman et al., 1991; U.S. EPA, 2016).

Atrazine is an herbicide that affects target plant species by interfering with the transfer of electrons from Photosystem II to Photosystem I (Jursinic and Stemler, 1983). Evidence suggests that non-target species, including amphibians, can be impacted by atrazine from chronic exposure at environmentally relevant concentrations (U.S. EPA, 2016). In the laboratory, atrazine exposure in amphibians has been observed to cause mortality and sublethal effects such as changes in physiological parameters at low concentrations. Mortality from acute atrazine exposure would be rare under typical exposure conditions because the LC50 (10,000≥ μg/L) for amphibians is much higher than observed in the field (Solomon et al., 2008; U.S. EPA, 2016). EPA’s recent risk assessment and weight of evidence analysis (U.S. EPA, 2016) found the potential for chronic risks to aquatic stage amphibians and ingestion-based risk concerns for herpetofauna.

Numerous studies have investigated impacts on amphibian development and health following atrazine exposure across multiple sub-lethal effects endpoints. The mode of action is not fully understood, however, and there is an ongoing debate and conflicting meta-analyses on its ability to significantly impact amphibian endpoints such as reproduction, growth, metamorphosis and predator avoidance (Solomon et al., 2008; Rohr and McCoy, 2010; Van Der Kraak et al., 2014; Bero et al., 2016) at environmentally relevant concentrations. Exposure to atrazine frequently alters biological parameters such as growth rate, timing of metamorphosis, and size at metamorphosis of larval amphibians (Rohr and McCoy, 2010). Confounding evidence exists on atrazine’s modulation of the timing of metamorphosis development (e.g. Choung et al., 2011; Coady et al., 2004; Kloas et al., 2009; Koprivnikar, 2010), likely because of the number of interacting processes in energetic pathways such as detoxification, growth, and alteration of development. Additionally, atrazine has been shown to decrease reproductive success, reduce immune function, and alter endocrine systems and behavior of amphibians. Much research has been done on the reproductive and endocrine disruption pathways (Hayes et al., 2002, 2010; Langlois et al., 2010), but less is understood about how energetics, growth, and metamorphosis pathways are disrupted physiologically. Metabolomics presents a pathway to link chronic, low dose stress from pesticides to sub-lethal effects on amphibian physiology at a cellular level before lethality.

While metabolomic biomarkers of atrazine exposure have been discovered in invertebrates (Ralston-Hooper et al., 2011a,b) and mammals (Lin et al., 2014), limited or no data are present using metabolomics to investigate the effect of atrazine on larval amphibians. However, few studies have used a more targeted approach to understand the effect of atrazine biochemically on growth and energy metabolism in amphibians. For example, Dornelles and Oliveira (2014) found decreased glycogen, total lipids, triglycerides, cholesterol, and total protein levels in gill, liver, and muscle with exposure to atrazine as well as increased lipid peroxidation in *Lithobates catesbeianus* larva. Zaya et al. (2011a,b) examined changes in gene expression to link atrazine exposure to changes in pathways converting lipids and proteins into energy and carbohydrate metabolism, fat storage, and protein synthesis in *Xenopus laevis*. Metabolomics was used to explore how the metabolome changes in early larval amphibian development and across all stages of frog development (Vastag et al., 2011; Ichu et al., 2014). Both studies found statistically significant changes in metabolite abundance corresponding to developmental stage. Changes in metabolomic pathway structure were also found in pathways including arginine and purine/pyrimidine, cysteine/methionine metabolism, and the urea cycle, among others.

In this study, two species of larval amphibians with contrasting life history strategies were used, *Hyla versicolor* (grey treefrog) and *Anaxyrus americanus* (American toad), to understand how atrazine impacts biochemical pathways. As adults, *H. veriscolor* are a more arboreal species most often encountered in deciduous forest canopy while *A. americanus* are more fossorial (Jensen, 2008). *A. americanus* and *H. veriscolor* both have a large range and are found throughout the Southeast in many land cover types (Kolozsvary and Swihart, 1999). During their breeding season both can be found in bodies of water which they utilize for depositing broods and remain through their larval stage. In their larval stage, they demonstrate developmental differences as well, for example hylids typically have a longer larval period and larger body size than bufonids at metamorphosis (Werner, 1986; Richter-Boix et al., 2011). Our objective is to understand how atrazine influences biochemical pathways of amphibians using metabolomics and support vector machine tools. We use gas chromatography coupled with mass spectrometry analysis to measure changes in endogenous metabolites. We investigate if metabolites influenced by atrazine can be used as biomarkers of exposure in amphibian larvae; and understand if the physiological changes induced by atrazine exposure are species specific in amphibian larvae.

## 2. Methods

### 2.1. Tadpole collection and rearing

To minimize the potential of previous pesticide exposure, American toad egg masses (*Anaxyrus americanus*) were collected from ponds within the Whitehall Forest research facility, part of the University of Georgia (UGA) in Athens, GA during March 2014. Grey treefrog (*Hyla versicolor*) tadpoles were collected from eggs that were laid in aerated artificial pools at the U.S. EPA Athens, GA. All eggs, tadpoles, and larvae were reared in aerated aquariums and fed fish food ad libitum after hatching. Housing, rearing, and animal studies were all done in accordance with approved animal use and care protocols.

### 2.2. Experimental design

All pesticide exposures were conducted with analytical grade pesticide active ingredients having purity ≥98%. Atrazine was obtained from the U.S. EPA’s National Pesticide Standard Repository in Fort Meade, MD. Atrazine was dissolved in methanol at a stock concentration of 250 mg/L and this stock was aliquoted in 50 mL centrifuge tubes for concentration levels of 10, 50, 250, and 1250 μg/L (0.046, 0.23, 1.16, and 5.80 uM). Concentrations were confirmed via liquid chromatography coupled with mass spectrometry in a one-time confirmation analysis. All residual methanol was evaporated under nitrogen prior to the addition of aged pond water for all exposures.

Gosner stage 22–24 tadpoles were exposed for 48 h in centrifuge tubes (4 individuals/tube) with no food added. Gosner stage was identified with microscopy. For each exposure class there were five replicate centrifuge tubes. Following exposure, tadpoles were euthanized, rinsed with milliQ water and placed individually in a 2 mL centrifuge tubes for metabolite extraction (see below). All tadpoles and metabolomic samples were stored <1 year at −80 °C until analysis.

### 2.3. Tissue preparation

Whole tadpole bodies were extracted using a mixture of methanol, deionized water (DiH2O), and chloroform (CCl3) to a final volume of 1185 μg following previously published methods (Viant, 2007). A 3.2-mm stainless steel bead (Biospec Products, Inc., Bartlesville, OK) was added to each centrifuge tube and homogenization was performed in a tissuelyser (Qiagen, Valencia, CA). Each metabolite-containing phase (polar and non-polar) was removed with a pipette and aliquoted into GC vials prior to lyophilization. Samples were sequentially derivatized with 30 μL *O*-methoxyamine-HCL dissolved in pyridine (Supelco, Bellefonte, PA), with a final concentration of 20 mg/mL, and 50 μL BSTFA + TMCS (N,*O*-bis(trimethylsilyl)trifluoroacetamide with 10% Trimethylchlorosilane, Thermo Scientific, Bellefonte, PA). After derivatization, polar samples were transferred to microtarget inserts, placed in GC vials, and analyzed within 72 h of derivatization completion.

### 2.4. GC/ToF-MS analysis

Samples were analyzed on a Leco Pegasus 4D Tof-MS interfaced with an Agilent 7890B gas chromatograph (Agilent Technologies, Santa Clara, CA) and equipped with a multipurpose sampler (Gerstel Inc, Linthicum, Maryland). Chromatographic separation was achieved on a DB-5MS (30 m × 0.25 mm, 0.25 μm, Agilent Technologies, Santa Clara, CA) with an initial oven temperature of 60 °C held for 2 min and ramped at 80/min to 300 °C with a hold time of 5 min. Helium was used as the carrier gas and maintained at a constant flow on 0.8 mL/min. Samples (2 μL) were injected in splitless mode with an injector temperature of 275 °C. The transfer line temperature and ion source temperatures were 280 °C and 225 °C, respectively. Mass spectra were acquired with a scan range of 50–650 *m*/*z* at an acquisition rate of 20 spectra/s.

### 2.5. Statistical analysis

**Fig. 1:**
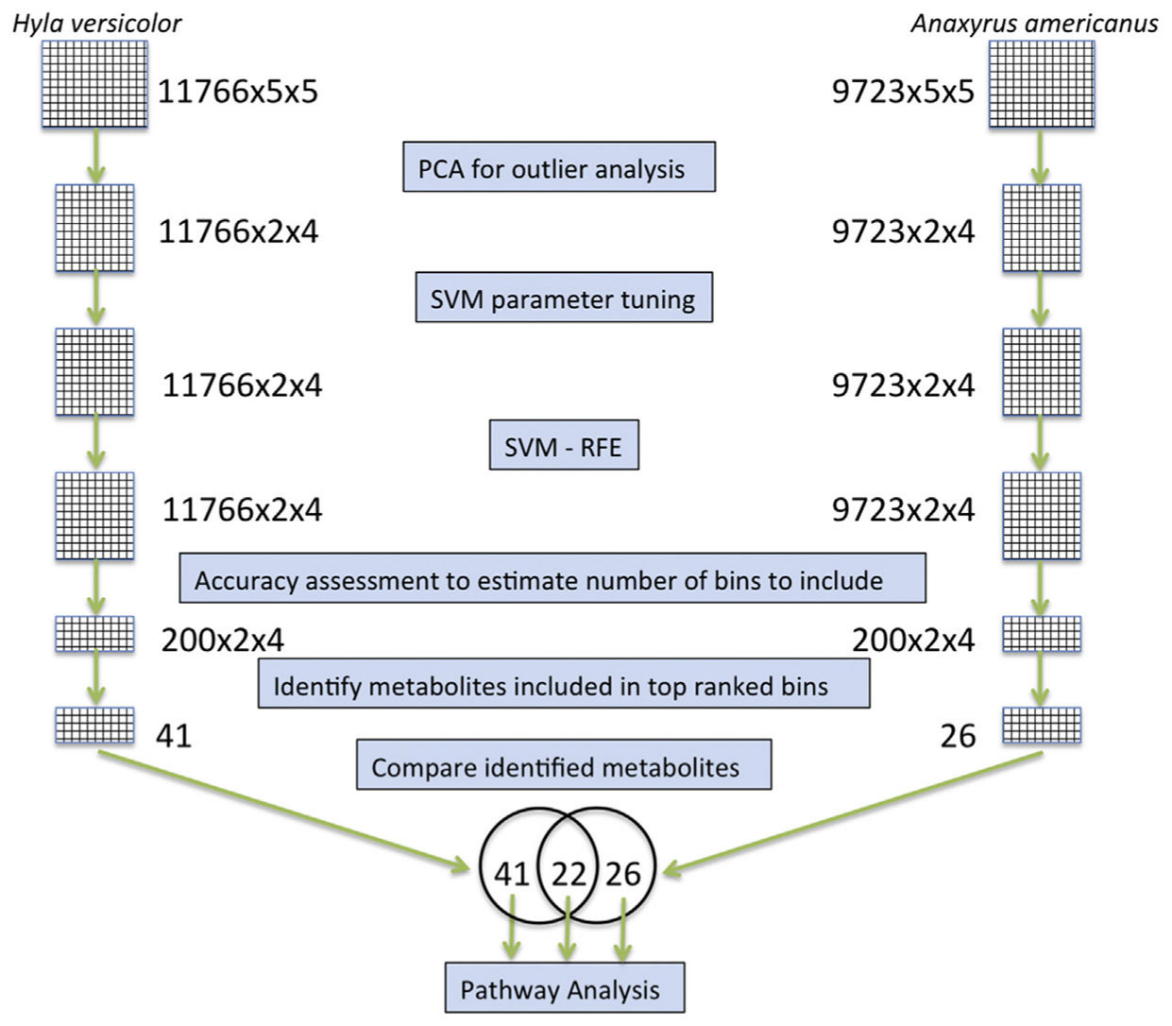
Flow diagram of post-processing data analysis steps (PCA all, for high concentration versus control). The 22 identified metabolites found in common between *H. versicolor* and *A. americanus* are referred to as the biomarker subset.

To account for possible variability in retention time of mass fragment spectra (*m*/*z*) from chromatographic peaks across individual GC–MS runs, XCMS Online was used to pre-process the raw data (Tautenhahn et al., 2012). This included correction alignment based on retention time and similarity of mass fragment ions to produce binned data organized by mass fragment abundance at each time point. Pre-processing included noise reduction as well. For multivariate tests, abundance values were univariate scaled and normalized to constant sum to account for variability in peak size as well as relative abundance of metabolites. To detect outliers, principal components analysis was used (Stacklies et al., 2007). All the treatments were used for pre-processing (Fig. 1) and multivariate outlier analysis. Two metabolomic profiles were considered outliers in *H. versicolor* and one profile in *A. americanus* based on geometric distance outside of a 95% confidence interval in the PCA score plots.

Support vector machine (SVM) methods with recursive feature elimination (RFE) were applied to the metabolomic profiles produced once outliers were eliminated (e.g. Guan et al., 2009). SVM creates a high-dimensional non-linear boundary for classification purposes by creating linear boundaries in a transformed dimensional space. In this case, the classifications are simply the control and the high exposure treatments. RFE ranks bins in the metabolomic profiles in order of importance to the classification. The variable rankings from the RFE distinguish the peaks most responsible for driving the classification and help differentiate differently expressed metabolomic biomarkers of exposure from un-induced biological variability in the samples not related to the toxicity process. Reducing the number of variables (i.e. metabolite bins) for building classification models can increase classification accuracy and increase the interpretability of the results. To distinguish peaks driving differences between control tadpoles and tadpoles exposed to atrazine, metabolomic profiles from the highest treatment (1250 μg/L) were established and compared to the control treatment with SVM-RFE. A radial non-linear kernel was used in all the SVM classifications while the cost and gamma parameters were optimized between 1 × 10^−11^ and 1 × 10^11^. Bootstrapping with repeated three fold cross validation to assess classification accuracy was used in these models. Statistical analysis was performed using R 3.0.1 statistical software (R Core Team, 2016) with the MUMA package (Gaude et al., 2013) and e1071 (Hornik et al., 2006).

The 200 top-ranked retention time mass fragment bins were putatively identified separately for each species. To tentatively identify the detected metabolites, peaks were searched in the National Institute of Standards and Technology (NIST) library spectral database with the available ChromaTOF software (LECO Corporation, St. Joseph, MI). Metabolites were identified by comparing retention time (>0.05 s) and mass spectrum to those in the NIST 14 database. Each selected and identified metabolite was assigned a similarity value (SV) to the NIST spectra; SV values >700 were considered a positive putative identification.

Metabolites identified from the 200 top-ranked retention time mass fragment bins were compared between *A. americanus* and *H. versicolor*. Of those 200, the subset of metabolite bins perturbated by atrazine in both *H. versicolor* and *A. americanus* will be referred to as biomarkers in this study. To examine how well biomarkers can distinguish an exposed tadpole from a control, we separately ran a support vector machine model classification with just identified biomarkers on the control tadpoles and each one of the concentrations. To test prediction performance, three-fold cross-validation of average classification accuracy across 250 repeated simulations was used. Models were first tuned and then repeated three-fold cross-validation to assess classification accuracy.

Metabolites from the 200 top-ranked bins and biomarkers were then associated with biochemical pathways using MetaboAnalyst 3.0, a web application that uses the KEGG metabolomic pathways for the pathway knowledgebase. These pathways represent different potential mechanisms of action of the stressors. They are ranked by the statistical likelihood that perturbated metabolites are found at a greater than random chance (p < 0.05), with correction for multiple comparisons, as well as their potential impact to the pathway based on its location and topology. We report pathways with more than three putatively identified metabolites resulting from the 200 top-ranked bins, as ranked with SVM-RFE.

## 3. Results

### 3.1. Metabolites

To assess changes in metabolic pathways in larval amphibians induced by atrazine, a metabolomic analysis of polar metabolites for *A. americanus* and *H. versicolor* was performed. Survival of amphibians was 100% across all treatments. In *A. americanus*, 11,766 retention time matched mass fragment bins were identified in pre-processing and used for temporal alignment, normalization and scaling using XCMS. Similarly, in *H. versicolor*, 9723 bins were identified. All exposure treatments were used for pre-processing and multivariate analysis as described above (Fig. 1).

**Fig. 2:**
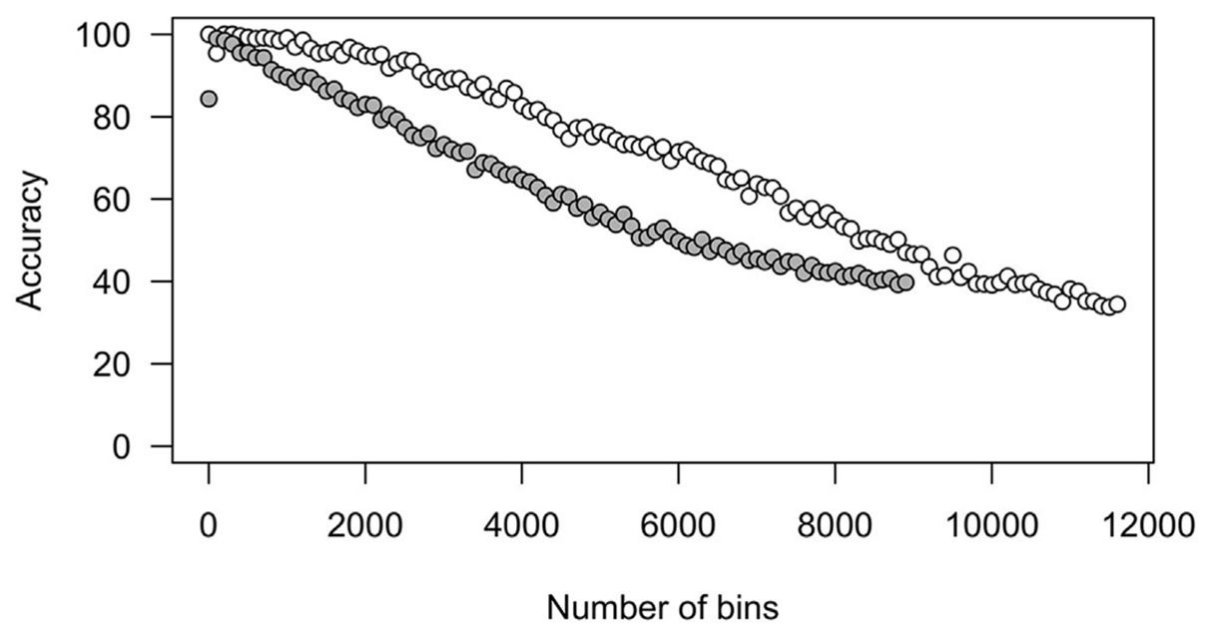
Cross-validation and classification accuracy over increasing number of ranked bins included in the support vector machine classification for *H. versicolor* (grey) and *A. americanus* (white).

SVM models were then applied with and without RFE to determine whether atrazine caused changes in the metabolomic profile of exposed and non-exposed tadpoles. When comparing the spectral response from the highest concentration (1250 μg/L), to the controls, only the SVM model demonstrated low classification accuracy values of <50%. However, by limiting the number of retention time mass fragment abundance bins input into the classification model by rank importance using RFE, accuracy increased to >90% (Fig. 2). Classification accuracy was greater than 80%, with 1–2200 bins (<1%–22%) included for *H. versicolor. A. americanus* SVM classifications were analyzed independently from *H. versicolor*, and utilizing feature-ranked bins similarly affected classification accuracy of the SVM (Fig. 2). Comparing control and atrazine exposed tadpoles (1250 μg/L), *A. americanus* classification accuracy increased to 100% with inclusion of fewer bins, and decreased to <80% after more than 4300 bins were included.

To investigate the metabolites driving the differences in metabolomic profiles of control tadpoles and tadpoles exposed to 1250 μg/L atrazine, the top 200 ranked retention time mass fragment bins from the SVM-RFE classification model were identified. Of the 200 top-ranked retention time peak bins, 67 metabolite peaks were identified and 26 (39%) of them could be putatively identified by database searching for *A. americanus*. For *H. versicolor*, 66 metabolite peaks were found to correspond to the 200 top-ranked retention time peak bins and 45 (68%) could be putatively identified by the NIST library (Table 1). The analysis identified amino acids (including methionine, serine); mono- and disaccharides (including maltose, glucose, galactose); fatty acids (including dicarboxylic and carboxylic acids); heterocyclic aromatics (e.g., purine); purine nucleosides (including guanosine); imidazoles (creatinine); ureas; and cyclic polyalcohols such as *myo*-inositol. Some mass fragment peaks were detected that could not be identified by retention time and mass spectra with available standards and are possibly amphibian-specific metabolites, further amphibian metabolome characterization is needed to address these peaks.

### 3.2. Biomarker analysis

**Fig. 3:**
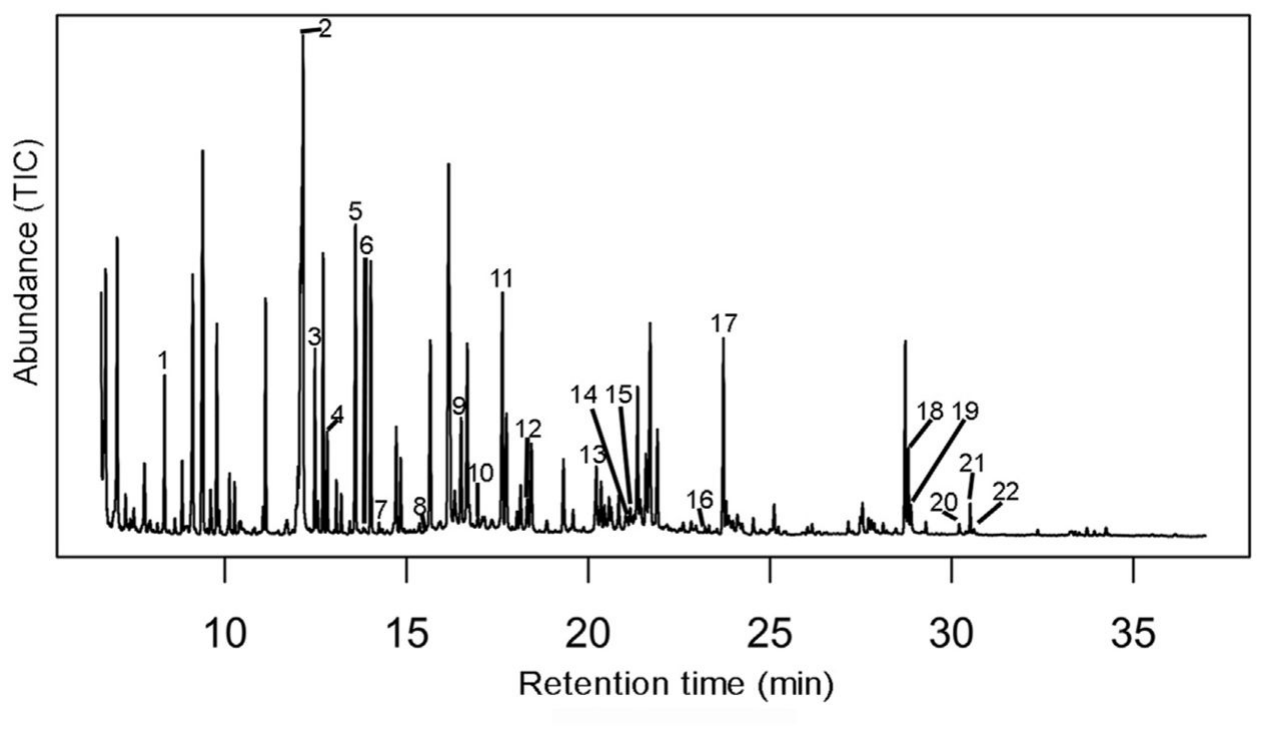
A GC–MS chromatogram of a control Gosner stage 22 *Hyla versicolor* larvae. 1: Lactic acid, 2: Phosphate, 3: Glycine, 4: Succinic acid, 5: Serine, 6: Threonine, 7: Pyrimidine, 8: Proline, 9: Creatinine, 10: Glutaric acid, 11: Glutamic acid, 12: Ribose, 13: Purine, 14: Glucose, 15: Galactose, 16: Palmitic acid, 17: Myoinositol, 18: Inosine, 19: Adenosine, 20: Maltose, 21: Guanosine, 22: Stearic acid.

To develop potential biomarkers of exposure, the metabolites identified from the 200 top-ranked retention time mass fragment bins from each species (Fig. 1) were compared. Twenty-two metabolites were identified as being affected by atrazine in exposure in both *A. americanus* and *H. versicolor* metabolomes (Table 1) including amino acids, short chain acids, purine nucleosides, monosaccharides, and fatty acids (Fig. 3).

**Fig. 4:**
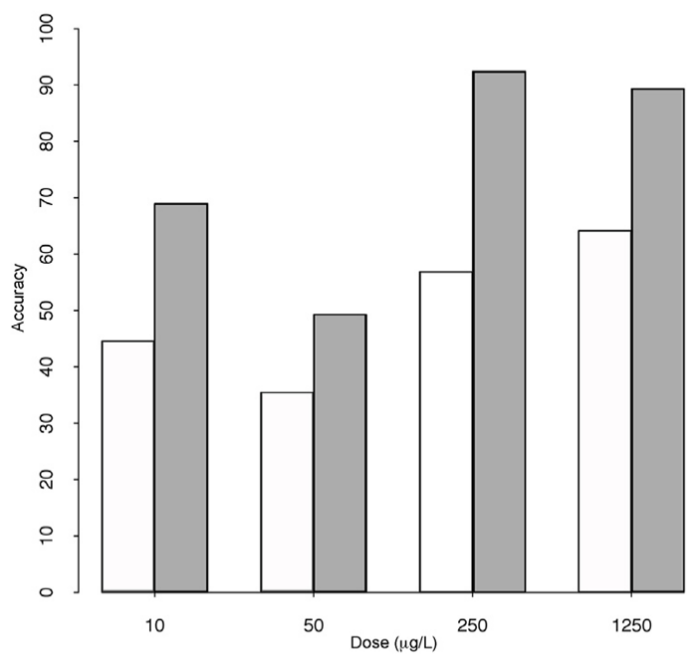
SVM models were used to classify metabolomic profiles using the biomarker subset determined by the overlap in identified endogenous metabolites from the 200 top-ranked retention time mass fragment bins between *A. americanus* (white) and *H. versicolor* (grey). Percent accuracy is the classification rate of the atrazine concentration (10, 50, 250, and 1250 μg/L) compared to the unexposed control.

The classification accuracy of biomarkers developed from the 1250 ug/L concentration with lower exposure concentrations (10, 50, and 250 μg/L) was tested (Fig. 4). Classification accuracy is the mean percentage of times an SVM model assigns the metabolic profile to the correct exposure concentration in 250 repeated threefold cross-validations, using the biomarker subset. Biomarker analysis of *H. versicolor* tadpoles at lower exposures had classification accuracies ranging from 49 to 92%. Biomarkers of exposure identified by comparing control tadpoles to tadpoles exposed at the highest concentration were most effective at classifying tadpoles exposed at 250 ug/L concentration (92%). Interestingly, *A. americanus* tadpoles had lower classification accuracies than *H. versicolor* tadpoles, ranging from 35 to 64%.

### 3.3. Pathway analysis

Pathways with more than three identified metabolites resulting from the top-ranked bins, ranked with SVM-RFE, for *A. americanus*, *H. versicolor*, and the biomarker subset are shown in Table 2. Amino acid metabolism pathways were commonly found to be impacted. For *A. americanus*, the pathways most likely to be significantly affected, based on overlap between perturbated metabolites and pathways, are: pentose phosphate; aminoacyl-tRNA biosynthesis; galactose metabolism; glycine, serine and threonine metabolism; and purine metabolism. For *A. americanus*, Holmes adjusted *p* values indicated no pathway was significantly impacted. While for *H. versicolor*, pathways with more than three metabolites, and with Holmes adjusted *p* < 0.05, include aminoacyl-tRNA biosynthesis, nitrogen metabolism, cyanoamino acid metabolism, alanine, aspartate, and glutamate metabolism; glycine, serine, and threonine metabolism, arginine and proline metabolism; and purine metabolism. Pathways most likely to be impacted from the biomarker subset based on more than three found metabolites include aminoacyl-tRNA biosynthesis; purine metabolism; and glycine, serine, and threonine metabolism. Although pathways including more than three metabolites for the biomarker subset were found, the Holmes adjusted *p*-values were not significant (*p* > 0.05).

**Fig. 5:**
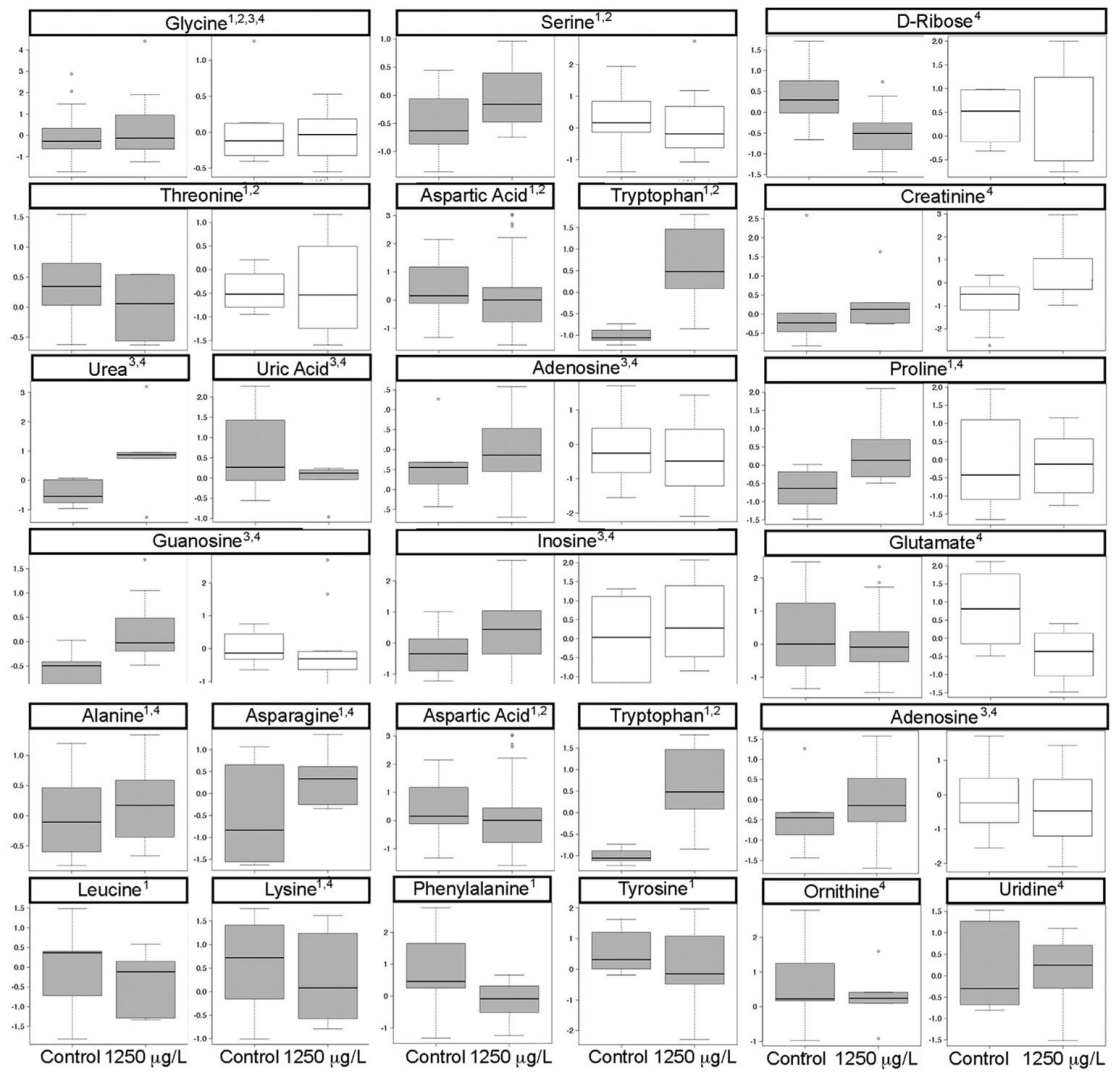
Box plots of impacted endogenous metabolites in the (1) aminoacyl tRNA biosynthesis pathway, (2) glycine, serine, and threonine metabolism pathway, (3) purine metabolism pathway, (4) purine/pyrimidine/arginine/urea pathway illustrated in Ichu et al. (2014). Relative abundance values are scaled and normalized for *Hyla versicolor* (grey) and *A. americanus* (white).

Boxplots of the metabolites in the top three indicated pathways for the biomarker subset demonstrated perturbations in measured metabolites, as well as variability in directional response across species. Direction does not necessarily indicate an increase or decrease in demand for that metabolite in the biochemical pathway, however, it could indicate inhibition or activation of the enzyme that catalyzes synthesis of the metabolite. Tryptophan which is utilized in glycine, serine, and threonine metabolism, as well as aminoacyl tRNA biosynthesis, demonstrates large increases with atrazine exposure of 1250 ug/L (Fig. 5). In purine metabolism, the majority of identified metabolites indicate a positive fold change (Fig. 5). Purine nucleosides (adenosine, guanosine, and inosine) increase with atrazine exposure in *H. versicolor*, although the directional response is varied in *A. americanus* (Fig. 5). Proline, threonine and glycine, and amino acids involved in the aminoacyl tRNA pathway all demonstrate a consistent direction of change in the two species.

## 4. Discussion

Statistical analyses of the acquired spectra demonstrated changes in endogenous metabolites between control and exposed tadpoles. Biochemical fluxes observed in the exposed group of both *A. americanus* and *H. versicolor* appeared in a number of classes of biochemical compounds, with the most frequently influenced groups being amino acids, sugar derivatives, fatty acids, and purine nucleotides. The 22 metabolites impacted in *A. americanus* and *H. versicolor* or the ratio of concentration fluxes could potentially be used as biomarkers of atrazine exposure. Purine metabolism; aminoacyl-tRNA biosynthesis; and serine, threonine, and glycine metabolism are the most commonly impacted biochemical pathways in both species.

One of the primary consequences of exposure to atrazine was perturbation of amino acid metabolism and specifically gluconeogenic amino acids. Amino acids that are important for distinguishing between control and atrazine exposure in SVM classifications for *A. americanus* include glycine, alanine, homoserine, and proline, as well as tyrosine and lysine for *H. versicolor*. Fluxes in amino acids suggest their utilization for energy production through proteolysis, the break-down of proteins to amino acids, conversion to ketones or glucose through gluconeogenesis for energy (Viant et al., 2006; Jones et al., 2008; Zaya et al., 2011b). Jones et al. (2008) found similar fluxes in amino acid metabolites and suggested a decrease in glycolysis and increase in gluconeogenesis because of increased amino acids in earthworms exposed to PAHs, along with a decrease in saturated fatty acids and changes in citric acid cycle intermediaries. Zaya et al. (2011a) found increased gene expression in *Xenopus* larva exposed to atrazine in pathways associated with conversion of lipids and proteins into energy, including metabolism of amino acids and decreased gene expression in protein synthesis and glycolysis/gluconeogenesis. In atrazine-exposed tadpoles, other energy-related metabolites (including glucose and galactose) were also higher, lending support to the hypothesis of impacts to energy metabolism. Additionally, a number of fatty acids including palmitic and stearic acid demonstrated fluxes in *H. versicolor* and *A. americanus* exposed to atrazine. Free fatty acids are an input to energy metabolism through the Krebs cycle. Fluxes in abundance may suggest that more or less energy is being created through the citric acid cycle during atrazine exposure. In this study, changes in amino acids, saturated fatty acids and sugars which, similar to the earthworms, suggest a perturbation of the tadpoles’ energy metabolism – perhaps an increase in gluconeogenesis and decrease in ATP creation mechanisms such as the glycolysis and citric acid cycle. Alternatively, a decrease in amino acids can be caused by starting to create defense proteins and repair mechanisms such as DNA repair enzymes (Spann et al., 2011).

Pathway analysis of individual species also supports the hypothesis that atrazine disrupts energy metabolism. In *H. versicolor*, nitrogen metabolism was found to be altered which could potentially increase the efficacy of protein catabolism. Prasad et al. (1991) suggests up-regulation of nitrogen metabolism in fish exposed to atrazine so that the fish could more efficiently use the end products of protein catabolism caused by atrazine. In *A. americanus*, two pathways involved in energy metabolism, pentose phosphate and galactose metabolism, were found to be impacted by atrazine exposure. Pentose phosphate metabolism creates precursors to nucleotide synthesis and is an energy metabolism pathway, which runs parallel to glycolysis-creating NADPH used in fatty acid synthesis (Lehninger, 2005). Galactose metabolism is the biochemical process of converting galactose to glucose to be used in energy metabolism (Lehninger, 2005).

Pesticide exposure can lead to induction of physiological response including greater energy demand to repair negative effects caused by stress. Changes in energy metabolism described by previous studies (Zaya et al., 2011a,b; Sanchez et al., 2008) may be related to increased demand for energy resulting from disruption of other pathways related to atrazine exposure such as oxidative stress, endocrine disruption, or altered, behavioral or immune response. Interestingly, the present study found purine metabolism to be impacted by atrazine in both species. Purine metabolism synthesizes and breaks down purines, creates enzymes involved in anti-oxidation, and is linked to energy metabolism via creation of AMP, GMP, etc. (Patkar et al., 2009; Slaninova et al., 2009). The data suggest that atrazine may influence purine metabolism through enzyme substrate similarity, or by increased energetic costs of detoxification generating oxidative stress. Enzymes contained in bacteria that break down atrazine belong to the amidohydrolase super family of proteins (Shapir et al., 2002). Enzymes in this family are utilized in purine metabolism to catalyze hydrolysis of cyclic C N bonds in nucleotide metabolism (Nam et al., 2005). One plausible mechanism for disrupting the purine metabolism pathway is that the amidohydrolase enzymes normally used in purine metabolism were more attracted to the atrazine surface and used to break down atrazine. Evidence includes increased abundance of adenosine and guanosine with atrazine exposure (Fig. 7).

Other studies have demonstrated oxidative stress as an effect of atrazine exposure on diverse organisms, using genomics or proteomics including invertebrates (Ralston-Hooper et al., 2011a,b; Song et al., 2009; Thornton et al., 2010); fish (Jin et al., 2010; Sanchez et al., 2008); and amphibians (Dornelles and Oliveira, 2014). Generation of free radicals during metabolism of atrazine has been linked to oxidative stress and the consequent alteration of purine metabolism. Studies have shown that atrazine can create oxidative stress by increasing the concentration of reactive oxygen species and damage caused by excess reactive oxygen species, as well as by influencing anti-oxidant enzyme activities. Dornelles and Oliveira (2014) found that lipid peroxidation levels increased in *Lithobates catesbeianus* tadpoles exposed to atrazine; lipid peroxidation is the degradation of lipids by reactive oxygen species. More research is needed to further understand the mechanism of action of atrazine in purine metabolism.

Fluxes in the aminoacyl-tRNA pathway could be correlated with an increase in demand for anti-oxidative enzymes, stress proteins, or DNA repair enzymes. Aminoacyl-tRNA biosynthesis builds amino-tRNA used for protein-building and has been linked to the nervous system, oxidative stress, and metabolic changes (Trushina et al., 2013). Atrazine exposure has been linked to higher levels of mRNA for genes encoding antioxidant proteins and perturbation of antioxidant enzyme activities (SOD and CAT) in zebrafish (Jin et al., 2010).

Ichu et al. (2014) described how metabolic profiles changed during amphibian larval development. Many of the altered metabolites that changed in tadpoles exposed to high levels of atrazine were also identified as dynamic based on larval stage. The highest overlap was in the urea cycle, arginine, and purine/pyrimidine metabolism pathway. Metabolites involved in purine metabolism, urea cycle, and arginine and pyrimidine metabolism have demonstrated significant abundance fluxes as frogs undergo metamorphosis (Ichu et al., 2014); the aforementioned pathways have many metabolites in common (Fig. 7). In this study atrazine exposed tadpoles demonstrated changes in a subset of the pathways that are involved in amphibian metamorphosis. Purine metabolism produces nucleotides and nucleosides, which are components of DNA and RNA, but are also involved in energy metabolism.

Additionally, in *H. versicolor*, tryptophan demonstrated a large increase in abundance, with exposure to 1250 ug/L atrazine. Lin et al. (2014) linked change in level of tryptophan to serotonin levels in the brain of mice. These results suggest that further study with tryptophan and intermediates in serotonin metabolism in amphibians could be useful to understanding possible neurological effects.

The machine learning technique used, SVM-RFE, was useful for distinguishing patterns in metabolomics with high biological variability. Metabolomics is a potentially more sensitive technique to examine changes in non-endocrine perturbations to larval physiology from atrazine. LOEC levels (200–10,000 μg/L) show a large range of sensitivity when utilizing traditional endpoints such as size, time to metamorphosis, and/or survival (Solomon et al., 2008; U.S. EPA, 2016). SVM-RFE’s ability to distinguish between exposed and non-exposed larva was effective for *H. versicolor* (>90%) at 250 μg/L, suggesting that metabolomic biomarkers could be a more sensitive endpoint for atrazine exposure than traditional toxicity assays.

The biomarker subset was more effective in classifying *H. versicolor* tadpoles than *A. americanus*, suggesting differences in species response. The metabolome of *H. versicolor* demonstrated higher perturbation than the *A. americanus* metabolome; we hypothesize that this suggests developmental rate and size at metamorphosis impacts larval metabolomic response to atrazine exposure. Additionally, different terrestrial-stage life history strategy could impact larval physiology. *A. americanus* is a more terrestrial species as an adult and, potentially, less susceptible to water loss through the skin than the arboreal *H. versicolor*. Terrestrial amphibian species have enhanced dehydration tolerance compared to more water-borne species. As adults, the primary method of foraging and locomotion has been related to capacity for aerobic and anaerobic metabolism. Inherent differences in aerobic metabolism capacity may drive differences in energetic metabolism perturbation by atrazine.

## 5. Conclusions

In conclusion, pathway analysis suggests that atrazine disrupts multiple metabolic pathways associated with energy and purine metabolism. Results from the current study contribute multiple lines of evidence that higher concentrations of atrazine (≥250 μg/L) impact energy metabolism of larval amphibians by diverting energy to detoxification, tissue repair, and restoring homeostasis. Purine metabolism, amino acid metabolism, galactose metabolism, and pentose phosphate metabolism are all pathways with increases or decreases in metabolite fluxes correlated with energy metabolism. Largely consistent with studies of other species, these data for an acute response time period (48 h) further support that high levels of atrazine impact energy metabolism. To determine the impact of atrazine on the metabolomics profile of larval amphibians over a chronic exposure period additional research is needed.

Metabolomic studies looking at different stressors and in various species have found perturbations in amino acid and energy metabolism. Other researchers have suggested this metabolomic signature pattern is part of an overall general stress response (Jones et al., 2008). Therefore, results from the current study likely embody a general stress response not restricted to atrazine, or even to amphibians. This study exposed larval amphibians to the active ingredient and not the formulation. More research is needed to understand effects of herbicide formulations with atrazine on amphibians however the generality of the response suggests it would also apply to herbicide formulations including atrazine. This implies that, while metabolomic profiling exhibits a high classification accuracy between control and high concentration levels of atrazine, they need further refinement to be useful in distinguishing between multiple stressors or other chemical stressors.

## Figures and Tables

**Table 1:** Endogenous metabolites putatively identified by the NIST library from 200 top-ranked feature bins for *Anaxyrus americanus* and *Hyla veriscolor* exposed to 1250 μg/L of atrazine. Pluses and minuses indicate change in relative abundance level of exposed, relative to control for the metabolite. Blanks indicate not detected in the top 200 bins. Twenty-two metabolites were identified as being jointly perturbed in both species.

Metabolite	*H. versicolor*	*A. americanus*	Metabolite	*H. versicolor*	*A. americanus*
Adenosine	+	−	Threonine	−	−
Butyric acid	+		Tryptophan	+	
Creatinine	+	+	Tyrosine	−	
Galactose	+	+	Linoleic acid		−
Glucose	+	+	Maleic acid	+	
Glutamic acid	−	−	Myoinositol	−	+
Maltose	+	+	Niacinamide	−	
Mannose		−	Ornithine	+	
Ribose	−	−	Palmitic acid	+	−
Gluconic acid		−	Phosphate	+	+
Glutaric acid	−	+	Phosphoric acid	+	
Glycine	+	+	Lactic acid	+	+
Guanosine	+	−	Pterin	+	
Inosine	+	+	Purine	+	+
Alanine	+		Putrescine	+	
Asparagine	+		Pyrimidine	−	+
Aspartic acid	−		Stearic acid	+	−
Leucine	−		Succinic acid	−	+
Lysine	−		Urea	+	
Phenylalanine	−		Uric acid	−	
Proline	+	+	Uridine	+	
Serine	+	+	Valeric acid	+	

**Table 2:** Metabolomic pathways affected by exposure to atrazine in *Anaxyrus americanus*, *Hyla versicolor*, and from the overlap of perturbated endogenous metabolites in both species. Pathways with more than three perturbated metabolites are shown.

Pathway	Total	Expected	Hits	Raw p	FDR	Impact	Holmes adjusted
***A. americanus***							
Pentose phosphate pathway	32	0.35	3	0.00	0.21	0.09	0.36
Aminoacyl-tRNA biosynthesis	75	0.81	4	0.01	0.21	0.06	0.61
Galactose metabolism	41	0.44	3	0.01	0.21	0.00	0.71
Glycine, serine and threonine metabolism	48	0.52	3	0.01	0.21	0.42	1.00
Purine metabolism	92	0.99	4	0.02	0.21	0.02	1.00
***H. versicolor***							
Aminoacyl-tRNA biosynthesis	75	1.25	12	0.00	20.80	0.00	0.00
Nitrogen metabolism	39	0.65	6	0.00	10.40	0.00	0.00
Cyanoamino acid metabolism	16	0.27	4	0.00	9.18	0.01	0.00
Alanine, aspartate and glutamate metabolism	24	0.40	4	0.00	7.51	0.04	0.01
Glycine, serine and threonine metabolism	48	0.80	5	0.00	6.91	0.08	0.02
Arginine and proline metabolism	77	1.28	6	0.00	6.56	0.11	0.02
Purine metabolism	92	1.53	6	0.00	5.64	0.26	0.04
Nicotinate and nicotinamide metabolism	44	0.73	4	0.01	5.20	0.40	0.05
Phenylalanine, tyrosine and tryptophan biosynthesis	27	0.45	3	0.01	4.66	0.68	0.08
Glutathione metabolism	38	0.63	3	0.02	3.73	1.00	0.19
Butanoate metabolism	40	0.66	3	0.03	3.59	1.00	0.20
Phenylalanine metabolism	45	0.75	3	0.04	3.29	1.00	0.25
Lysine degradation	47	0.78	3	0.04	3.18	1.00	0.26
Cysteine and methionine metabolism	56	0.93	3	0.06	2.74	1.00	0.34
***Biomarkers***							
Aminoacyl-tRNA biosynthesis	75	0.65	4	0.00	0.16	0.06	0.28
Purine metabolism	92	0.80	4	0.01	0.16	0.02	0.57
Glycine, serine and threonine metabolism	48	0.42	3	0.01	0.16	0.42	0.60
